# Respiratory Health and Cross-Shift Changes of Foundry Workers in Iran

**Published:** 2018-10

**Authors:** Mayam Saraei, Habibbolah Masoudi, Omid Aminian, Nazanin Izadi

**Affiliations:** Center for Research on Occupational Disease, Tehran University of Medical Sciences, Tehran, Iran

**Keywords:** Foundry, Pulmonary function, Respiratory symptom

## Abstract

**Background:**

Respirable dust exposure is associated with increased respiratory impairment. As there are various airborne contaminants in the foundry industry, our aim was to thoroughly examine the acute effects of ambient respiratory dust on the respiratory system.

**Materials and Methods:**

A cross-shift study was conducted in a cast iron foundry in Iran. A total of 200 participants, including 110 workers from production department and 90 office workers were enrolled in this study. Workers were evaluated with regard to respiratory symptoms using the American Thoracic Society (ATS) questionnaire and examination of their lung function by spirometry.

**Results:**

The mean exposure of all studied substances was higher than occupational exposure limits. The most common respiratory symptom in exposed workers was cough (24.5%). A statistically significant post shift reduction in FEV1 and FEF25-75 was seen in exposed group. After adjusting for age, working history, smoking and Body Mass Index (BMI), there was a significant decrease based on exposure in FEV1 and FVC.

**Conclusion:**

Dust exposure was a significant predictor of lung function. Implementing the health promotion program, periodic medical surveillance and efficient use of respiratory protection equipment could help to protect foundry workers from respiratory impairment.

## INTRODUCTION

It is well established that exposure to airborne contaminants in occupational industries increases the risk of developing respiratory diseases. Respiratory problems due to occupational exposures are influenced by the particle type, size, concentration, exposure duration and individual susceptibility. In addition to these factors, exposure to respiratory hazards varies according to the type of industry and also climate condition (1, 2).

The foundry produces metal castings through the process of pouring the metal in a mold, and removing the cast after the metal has solidified. There are several working stations in the foundry that include: melting, furnace, mold making, casting, cleaning and finishing (3).

Foundry workers are at risk of respiratory problems due to exposure to metal fumes, iron oxide, polycyclic aromatic hydrocarbons, gases, resins, isocyanates and dusts such as crystalline silica (4). These workers are at increased risk of respiratory impairment due to different exposures. Several studies have shown the association between foundry production and chronic effects on respiratory system, including chronic obstructive airway disease, pneumoconiosis, and cancers (5–10), but to the best of our knowledge and also the importance of adverse health effects of foundry workers, there are limited data about the acute effect of inhalation exposures abroad and especially in Iran.

One of the most important diagnostic methods for early detection of pulmonary dysfunction is spirometry (11). This study was designed to assess the acute effect of occupational exposures to respiratory hazards on the lung function of foundry workers. The effects of chronic exposure to airborne pollutants and associated factors on the lung function have also been examined.

## MATERIALS AND METHODS

This cross sectional study was carried out in Tehran, one of the provinces of Iran. This 40-year factory is one of the most important and experienced cast iron foundries in the eastern region of Tehran, which has been in continuous production of a wide range of standard cast iron grades, including: white, gray and ductile.

Exposed subjects had worked in different parts of the production line, including: furnace, sand casting, molding, and surface cleaning. Dust concentration at these working stations were measured with stationary air sampling pumps (SKC; 2 *L/min*) fitted with a Casella apex. The pumps had been previously calibrated before sampling. Exposure was measured by sampling total dust on polyvinyl chloride filters with a pore size of 5 *μm* placed in 37 *mm* open face holder. The pumps were put on 5 different parts of each working station. The mean of total dust in each section after 6 hours was analyzed quantitatively. The results of exposure assessment were compared with the Threshold Limit Value (TLV) of the American Conference of Governmental Industrial Hygienist (ACGIH) (12).

Two hundred subjects were enrolled in our study using random sampling based on the following criteria: male, age from 20–65 years old and at least 2 years work experience. One hundred and ten workers in the production process and 90 office workers were selected as exposed and non-exposed groups, respectively.

The participants were selected from those with no history of respiratory diseases such as asthma, bronchitis, emphysema, bronchiectasis, lung cancer…or any other chronic condition in the pre-employment assessment. The study was approved by the Ethics committee of the Tehran University of Medical Sciences. Participants were informed about the aim of the study.

Demographic and occupational characteristics, smoking habits and some questions about respiratory symptoms such as cough, sputum, dyspnea and chronic bronchitis (productive cough for two consequent years at least for three months) were recorded using an American Thoracic Society (ATS) standard questionnaire (13). An occupational medicine physician examined and evaluated all workers for respiratory problems. Pulmonary function tests were performed by an experienced technical staff using a calibrated spirometry apparatus (Model: spirolab MIR), in the morning before the start of work and at the end of work shift. Spirometric parameters including Forced Vital Capacity (FVC), Forced Expiratory Volume in one second (FEV1), FEV1/FVC, Peak Expiratory Flow Rate (PEFR), and forced expiratory flow at 25–75% of the FVC (FEF25-75) was measured in accordance with ATS criteria (14).

Data were analyzed by SPSS 22 (Chicago, IL, USA). Chi square test was used for determination of association between two qualitative variables, while independent sample t–test was used to evaluate the difference between quantitative variables both in exposed and non-exposed groups. Multiple linear regression analysis was used to evaluate the relation of spirometric indices to exposure while adjusting for age, working history (years employed at the factory), height, smoking and wearing masks. P value<0.050 was considered as the level of statistical significance.

## RESULTS

A total of 110 cast iron foundry workers as an exposed group were examined in the study along with 90 office workers as non-exposed group. Time Weighted Average (TWA) total dust and respirable dust concentrations in different parts of the factory are shown in table 1. All of the measurements exceeded the standards of the ACGIH.

**Table 1.** Eight-hour TWA (Time Weighted Average) total dust and respirable dust concentrations among different working stations

**Working stations**	Total dust*(nuisance) ***mg/m^3^***	Respirable dust** ***mg/m^3^***
**Furnace**	15.5	6.09
**Sand casting**	12.6	6.15
**Mold making**	11.8	6.02
**Surface cleaning**	10.32	5.33

* TLV (Threshold Limit Value)=10 *mg/m^3^*,

** TLV=5 *mg/m^3^*

The mean age was 39.1±8.9 ranging from 20–65 years. The exposed groups were significantly older and had lower education. The exposed population was found to have smoked more than the office workers (OR: 2.34). The difference in the mean Body Mass Index (BMI) and working history between two groups did not differ significantly (Table 2).

**Table 2.** Baseline characteristics of study population

**Variables**		**N (%)**	**Exposed Group** **N (%)**	**Un-exposed group** **N (%)**	**P value** **OR (CI95%)**
**Educational level**	< Diploma	55(27.5)	45(81.8)	10(18.2)	0.000^€^
	≥Diploma	145(72.5)	65(44.8)	80(55.2)	5.5(2.5–11.8)
**Smoking**	Yes	72(36)	49(68.1)	23(31.9)	0.005^€^
	No	126(64)	61(47.7)	67(52.3)	2.34(1.27–4.28)
**Wearing mask**	Yes	64(58.2)	64(58.2)	-	
	No	45(40.9)	45(40.9)	-	
**Age (mean±SD)**		39.15±8.09	40.8±9.17	37.11±8.16	0.003 *
**Working history (mean±SD)**		10.83±7.16	10.84±5.66	10.83±8.21	0.98 *
**BMI (mean±SD)**		26.85±4.5	26.39±4.5	27.41±4.41	0.11*

€ X^2^,

* Test for independent sample

It was shown that cough was the most frequent symptom in the exposed group (24.5%). Fifty-five (55.6%) of exposed group who had respiratory complaints were used to wearing masks in comparison with 44 (44.4%) who had no protection (p value 0.094). The lung function values for FVC and FEV1 were significantly lower for the exposed group (Table 3).

**Table 3.** Frequency of respiratory symptoms and mean values of pulmonary function tests in exposed and the unexposed subjects

**Spirometric parameters**	**Exposed Group**	**Un- exposed Group**	**P-value**
**FVC**	4.4±0.76	4.84±0.91	<0.001*
**FEV1**	3.49±0.64	3.80±0.56	<0.001*
**FEV1/FVC**	78.61±9.39	78.36±10.27	0.86*
**FEF**_**25-75 **_	3.31±1.04	3.60±0.98	* 0.48
**PEF**	8.45±1.65	13.38±42.7	0.22*
**Cough N(%)**	27(24.5)	12(13.3)	0.046£
**Dyspnea N(%)**	19(17.3)	9(10)	0.14£

€ X^2^,

* Test for independent sample

The percentage of cross-shift changes in the spirometric indices for FEV1 (140 m/s) and FEF25-75 (150 *ml/s*) differed significantly between two groups (Table 4).

**Table 4.** Mean changes from the before shift values of the Forced Vital Capacity (FVC), Forced Expiratory Volume in 1 s (FEV1), Peak Expiratory Flow (PEF) and Forced Expiratory Flow rate at 25%–75% of Forced Vital Capacity (FEF25-75) and after shift values in exposed group

**Spirometric parameters**	**Before shift**	**After shift**	**t**	**P-value**
**FVC**	4.93±0.75	4.4±3.5	1.62	0.10
**FEV1**	3.62±0.61	3.48±0.63	7.8	0.000
**FEV1/FVC**	78.96±6.32	78.6±9.39	0.54	0.58
**FEF**_**25-75**_	3.46±1.01	3.31±1.03	3.01	0.003
**PEF**	8.52±1.67	8.45±1.65	0.67	0.52

Pair sample t-test

However, the interaction between exposure and age was significant only for FEV1. Using multi linear regression analysis, exposure was still a significant predictor for FEV1 and FVC after adjusting for age, working history as continuous variables, and smoking, wearing masks and exposure as categorical variables (Table 5).

**Table 5.** Predictors of lung function tests in multiple regression analysis

**Covariates**	**FVC**		**FEV1**		**FEF 25-75**		**FEV1/FVC**	
	*B*	*P value*	*B*	*P value*	*B*	*P value*	*B*	*P value*
**(Constant)**	5.940	0.165	−2.491	0.019	−2.887	0.140	61.725	0.002
**Age**	−2.100	0.104	−0.016	0.004	−0.021	0.051	−0.024	0.822
**Height**	−0.013	0.000	0.039	0.000	0.039	0.000	0.077	0.485
**Working history**	0.045	0.273	0.000	0.961	0.011	0.391	0.085	0.533
**Smoking**	−0.011	0.384	0.170	0.032	0.413	0.005	2.62	0.081
**Wearing masks**	−0.098	0.448	−0.102	0.203	−0.194	0.192	−0.651	0.669
**Exposure**	−0.087	0.000	−0.318	0.001	−0.275	0.101	0.385	0.823

## DISCUSSION

The workers at the cast iron foundries are exposed to dust, fumes, and solvents comprising silica, carbon, iron oxide, lead oxide, manganese, toluene and benzene (6). Most of these airborne contaminants have been shown to have an adverse effect on the pulmonary function of exposed workers (15). In our study, cast iron foundry workers were found to have been exposed to higher concentrations of respirable dust than non-exposed group; furthermore, the measured respirable dust was higher in the sand casting section. On the other hand, office workers as the non-exposed group did not have any exposure to airborne contaminants, although 58.2% of exposed group normally wear protective masks. The workers who normally wear protective masks have lower frequency of respiratory symptoms (16). We found no association between wearing masks and respiratory complaints. It may be due to inappropriate mask type (simple instead of filtering-face piece respirators), insufficient and irregular use of the protection. According to the survey findings, there are potentially inadequate respiratory protection programs in metal industries (17). It is the obligation of each organization to evaluate its own respiratory protection program with respect to the Occupational Safety and Health Administration (OSHA) standards (18). Therefore, regular use of high quality personal protective equipment is recommended.

A reduction in the FEV1 and FVC was observed in the exposed group, indicating that occupational exposure to respirable dusts in the cast iron foundry workers has an important role in decreasing lung function. Our findings were in line with previous studies. Low reported lower mean values of FVC among steel foundry workers (19). Johnson found a significant decrease in FEV1 among iron and steel foundry workers (9). Similar effects have been found among 55 foundry workers and there was an inverse relation to dust exposure in a foundry plant and FVC (20).

We found that exposed workers have a higher frequency of respiratory symptoms including cough. Other studies in foundry workers showed similar results with an excess frequency of chronic bronchitis in exposed subjects (21, 22).

In the present study, we found a cross-shift reduction in FEV1and FEF25-75 among the exposed group. Our findings are in agreement with the result of Zarei et al. study among 55 foundry workers who found a significant association between exposure to silica dust and decreases in FVC values after shift reduction in FVC, FEV1, and FEF 25–75% values (20). To the best of our knowledge, too little attention has been paid to determine the acute effect of respirable dust on lung function parameters of foundry workers in literature. This phenomenon shows the short effect of respiratory hazards in cast iron foundry workers. This finding is in accordance with the results of an experimental study that mild thickening of alveolar wall was seen from 8 hour exposure and moderately severe in rat lung after 24 hour exposure (23).

The multivariate linear regression analysis of lung function parameters showed the clear effect of dust exposure on FVC and FEV1. The longitudinal and cross-sectional analysis of Myers’ study showed a reduction in FVC and FEV1 in relation with cumulative exposure (24). Therefore, the effect of smoking was not found as anticipated on FVC among the exposed group. The lower value of FEF25-75 and FEV1 was observed in smokers regardless of the effect of exposure. Smoking is one of the most important factors which adversely affects lung function. A reduction in FEV 1 and FEF25-75 is an indicator of obstructive pattern and small airway obstruction, respectively (25).

### Limitations

Our study was done on currently employed workers, therefore the healthy workers’ effect could have affected the results, when perhaps workers who had developed respiratory complaints were transferred or left the industry. Therefore, a follow up study or a comparative study of retired/resigned workers could reduce the healthy worker effect and better explain the association. Moreover, this cross sectional study was conducted in one factory, so the generalizability and causal association of our results is limited. Also due to insufficient sample size, we couldn’t compare different parts of the foundry processes.

## CONCLUSION

Results from the current study showed a significant relationship between dust exposure in foundry workers and cross shift reduction in lung values. Also, pulmonary function tests revealed some degree of impairment in comparison with office workers. According to this finding, the managers can use these results as a reference for future exposure and biologic monitoring and justifying regular health surveillance.

The existence of respiratory disorders and lung function impairment in foundry workers has raised serious concerns about their respiratory health. Therefore, implementing health promotion programs, more effective dust control measures such as ventilation and use of effective respiratory protective equipment, periodic medical examinations, including radiographic assessments, are recommended to control adverse respiratory effect of foundry workers.
